# Severe community-acquired pneumonia caused by *Chlamydia abortus* in China: a case report

**DOI:** 10.3389/fmed.2024.1426577

**Published:** 2024-07-22

**Authors:** Qiong-Fang Yang, Cai-Min Shu

**Affiliations:** Department of Respiratory Medicine, Affiliated Dongyang Hospital of Wenzhou Medical University, Dongyang, Zhejiang, China

**Keywords:** severe, community-acquired pneumonia, *Chlamydia abortus*, metagenomic next-generation sequencing, case report

## Abstract

**Background:**

*Chlamydia abortus* causes abortions in ruminants; it can also cause miscarriages and stillbirths in pregnant women. However, it rarely causes pneumonia in humans. Here, we report a case of severe community-acquired pneumonia caused by *C. abortus*.

**Case presentation:**

On admission to our hospital, a 74-year-old woman reported that she had had a fever, cough, phlegm in her throat, and shortness of breath for 10 days. In the local hospital, she was initially diagnosed with community-acquired pneumonia and treated with piperacillin–tazobactam for 4 days. However, her condition worsened, and she was therefore transferred to our hospital. On arrival at our emergency department, she was diagnosed with severe community-acquired pneumonia and treated with a high-flow nasal cannula and meropenem; she was then transferred to the Department of Respiratory Medicine. There, her condition continued to worsen despite continued treatment with the high-flow nasal cannula and omadacycline. After 24 h and emergency tracheal intubation, the patient was sent to the intensive care unit (ICU) for further treatment. The doctors in the ICU again adjusted the treatment, this time to meropenem along with mechanical ventilation; they also instituted methylprednisolone, ulinastatin, nadroparin calcium, and human immunoglobulin. In addition, bronchoalveolar lavage fluid was sent for metagenomic next-generation sequencing (mNGS). Subsequent mNGS suggested the presence of *C. abortus*, sequence number 5072; we therefore discontinued the meropenem and implemented a combination of doxycycline and moxifloxacin. After 8 days of treatment in the ICU, the patient’s condition improved; she was then extubated and, 3 days later, transferred back to the respiratory medicine department. The respiratory physician continued to administer doxycycline and moxifloxacin for 4 days, after which the patient was discharged with medication. A month later, a repeat computed tomography (CT) scan of the chest suggested that the lesions in both lungs had been largely absorbed.

**Conclusion:**

*C. abortus* can occasionally cause pneumonia in humans and, rarely, severe, life-threatening pneumonia. mNGS is uniquely suited for the early detection of this unusual infection. The combination of doxycycline and quinolones has been shown to be effective in severe pneumonia caused by *C. abortus*.

## Introduction

*Chlamydia*, as an obligate intracellular Gram-negative bacterium, is known to be responsible for several serious global healthcare challenges. Among these bacteria, *Chlamydia abortus* is an especially important zoonotic pathogen; it mainly causes infections in ruminants but, less frequently, may cause pneumonia in humans. Even more rarely, it can initiate an extremely severe pneumonia (1). Thus far, only six cases of pneumonia due to *C. abortus* have been reported worldwide; of these, five were not severe (2–6) and only one was severe (7). To further raise awareness of this rare disease, we here describe a case of severe pneumonia caused by *C. abortus*.

## Case presentation

**FIGURE 1:**
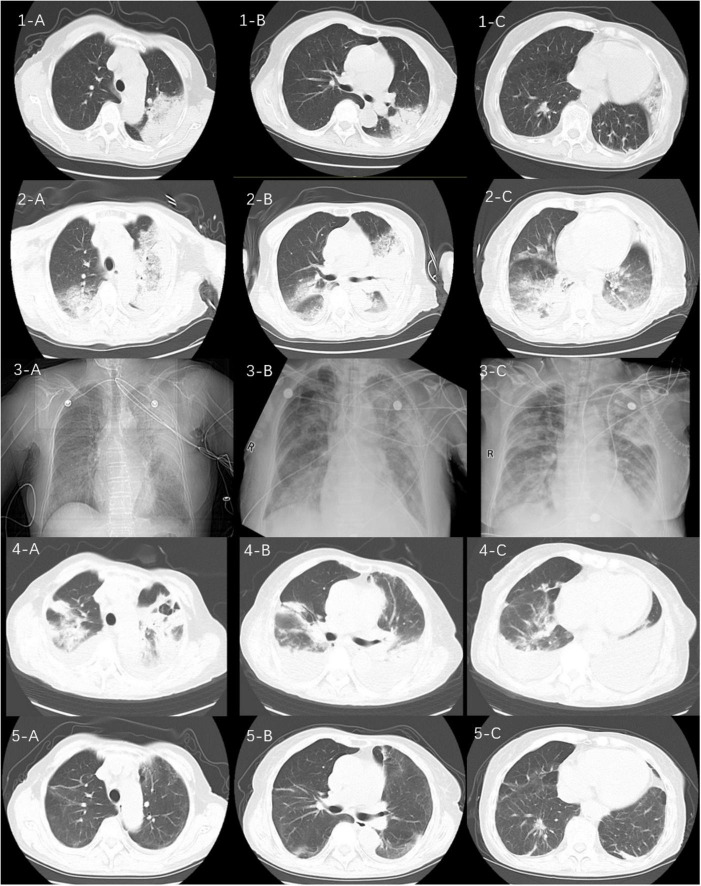
Sequential computed tomograms (CTs) of the patient’s chest. CT at a local hospital (1A–C) appeared to show a solid shadow in the upper lobe of the left lung. CT on admission (2A–C) showed multiple exudative lesions in both lungs. The next images (3A–C) represent the patient’s chest x-ray findings at the time of admission to our hospital, on the 5th and 10th days, respectively. CTs on the 15th day of admission (4A–C) suggested infection in both lungs with partially absorbed and partially enlarged lesions as well as an enlarging pleural effusion on the left side. CTs 1 month after discharge (5A–C) show substantial resorption of the lesions in both lungs.

A 72-year-old woman was admitted to our emergency department on December 1, 2023, with complaints of fever as well as cough, phlegm in the throat, and shortness of breath, all of which had been present for 10 days. She had had a cerebral infarction a year earlier as well as an aneurysm of the left internal carotid artery but did not take medication regularly. Nevertheless, she said that she was able to take care of herself. Ten days earlier, she had developed a cold and a fever with a temperature of about 38.0°C along with a cough, phlegm in her throat, dyspnea, generalized muscle aches and pains, and intermittent diarrhea. Her symptoms were not relieved by cold medicine. Thus, after 5 days of illness, she went to her local hospital. There, after computed tomography (CT) of the chest, she was diagnosed with community-acquired pneumonia. The CT pointed to an infection in the upper lobe of the left lung (Figure 1: 1A–C), and she was given piperacillin–tazobactam (4.5 g q8h as an intravenous infusion) for 4 days. However, her condition did not improve. She was then admitted to the emergency department of our hospital.

On the patient’s arrival at our emergency department, physical examination showed the following: temperature, 38.9°C; respirations, 26/min; pulse, 102/min; blood pressure, 130/75 mmHg; oxygen saturation, 72% (under mask oxygen of 4 L/min); clear consciousness; tachypnea; rhythmic dry and wet rales audible in both lungs; abdominal tenderness; and no swelling of the limbs. Laboratory tests showed the following: calcitonin, 9.0 ng/mL; C-reactive protein, 299 mg/L; leukocyte count, 11.12 × 10^9^/L; neutrophil ratio, 96.4% (which was significantly higher than normal). Arterial blood gas analysis showed type I respiratory failure and an oxygenation index of 108 mmHg. Blood biochemistry suggested hyponatremia and hypochlorhydria. Blood creatinine, liver enzymes, and lactate dehydrogenase were significantly higher than normal. The levels of plasma pro-brain natriuretic peptide (pro-BNP) were 1150 pg/mL (Table 1). A repeat CT scan of the chest suggested infectious lesions in both lungs and a small pleural effusion on each side (Figure 1: 2A–C). Color Doppler ultrasound of the vasculature of both lower extremities revealed atherosclerosis and intermuscular venous thrombosis of both lower legs. Color Doppler ultrasound of the heart showed aortic arteriosclerosis. Tricuspid valve regurgitation was mild; the ejection fraction was 65%. The patient was diagnosed with severe community-acquired pneumonia and was ventilated by a high-flow nasal cannula (oxygen concentration, 80%; flow rate, 55 L/min) with the oxygen saturation maintained at about 95%; she was also medicated with meropenem (0.5 g q8h by intravenous infusion) and methylprednisolone (40 mg q12h by intravenous infusion). At one point during this procedure, her blood pressure dropped to 90/52 mmHg; however, it returned to the normal level after active fluid replacement.

**TABLE 1:** Changes in laboratory indicators.

Test	Day 1	Day 3	Day 6	Day 9	Day 14	Day 18	Reference range
WBC	11.12	15.06	7.01	7.94	6.15	4.40	3.5–9.5 (10^9^/L)
CRP	299.3	171.8	53.4	29.5	14.8	22.8	<8 (mg/L)
PCT	9.01	7.25	1.54	0.526	0.497	0.385	<0.1 (ng/ml)
IL-6	404.71	57.18	–	–	4.18	–	<5.4 (pg/ml)
D-dimer	4.52	5.55	–	2.66	2.65	12.79	<0.5 (ug/ml)
Cr	114	122	110	74	–	108	57–111 (umol/L)
Pro-BNP	1150	–	4102	1400	–	279.5	5–125 (pg/ml)
ALT	461	405	308	74	64	32	15–40 (U/L)
AST	72	217	195	36	40	13	9–50 (U/L)
Na	126.0	137.1	136.0	129.0	135.0	137.3	137–147 (mmol/L)
Cl	87.1	104.0	111.0	102.0	100.0	99.9	99–110 (mmol/L)
Alb	20.3	–	26.6	–	28.5	37.8	40–55 (g/L)
LDH	539	468	–	–	–	253	109–245 (U/L)

WBC, White blood cell count; CRP, C-reactive protein; PCT, Procalcitonin; interleukin-6, IL-6; Cr, creatinine; Pro-BNP, Pro-Brain Natriuretic Peptide; ALT, alanine aminotransferase; AST, aspartate aminotransferase; Na, serum sodium; Cl, serum chlorine; Alb, Albumin; LDH, lactate dehydrogenase.

The next day the patient was admitted to the respiratory ward, and ventilation by high-flow nasal cannula (oxygen concentration 75%, flow rate 50 L/min) was continued to maintain oxygen saturation at about 95%. The retest of her blood inflammation index was still significantly higher than normal (Table 1), and it was felt that the atypical pathogen infection had to be covered. The treatment was therefore adjusted to omadacycline (0.1 g qd by intravenous infusion with 0.2 g being given on the first day), methylprednisolone (40 mg q12h by intravenous infusion), ambroxol hydrochloride (30 mg bid by intravenous infusion) for phlegm, and nadroparin calcium (4100 U qd by hypodermic injection) for anticoagulant and symptom-supportive therapy.

To detect the etiology of her pneumonia, the patient underwent a series of microbiologic diagnostic tests for bacteria, fungi, and viruses, and including staining and culture, serologic testing, and others. However, all of the tests were negative (Table 2). Thus the patient’s clinical symptoms continued to deteriorate, with oxygen saturation maintained between 89 and 95%. She experienced shortness of breath and—after being given an emergency tracheal intubation for mechanical ventilation 24 h after admission—was transferred to the ICU.

**TABLE 2:** Etiological examination results.

Test	Results	Reference range
Blood culture	Negative	Negative
Sputum bacterial culture	Negative	Negative
Cryptococcus antigen	Negative	Negative
T-SPOT	Non-reactive	Non-reactive
Fungus-D glucan determination	<40	<60 (Pg/ml)
Galactomannan determination	0.2	<0.25 (ug/L)
Sputum smear for acid-fast bacilli	Negative	Negative
Sputum tuberculosis culture	Negative	Negative
Influenza virus nucleic acid	Negative	Negative
COVID-19 nucleic acid	Negative	Negative
Adenovirus nucleic acid	Negative	Negative
Respiratory syncytial virus nucleic acid	Negative	Negative
Mycoplasma pneumoniae nucleic acid	Negative	Negative
Chlamydia nucleic acid	Negative	Negative
Legionella pneumophila nucleic acid	Negative	Negative
Streptococcus pneumoniae nucleic acid	Negative	Negative
Haemophilus influenzae nucleic acid	Negative	Negative
Bordetella pertussis nucleic acid	Negative	Negative

At the ICU, her treatment was adjusted to meropenem (1.0 g q8h by intravenous infusion) with continuing methylprednisolone, ambroxol hydrochloride, and nadroparin calcium plus the addition of thymopeptide (1.6 mg qd by hypodermic injection) and immunoglobulin (10 g qd by intravenous infusion) to improve the immunity. On the next day, bedside bronchoscopy showed that there was more white mucous sputum in both bronchi, and the lumen was clear after aspiration. The bronchoalveolar lavage fluid was then sent for mNGS examination. Two days later, the result suggested *C. abortus*, with 5072 sequence numbers and a relative abundance of 95.03%. A repeat bedside radiograph of the chest showed infection in both lungs, with greater infection in the right lung and partial absorption of infection in the left lung by comparison with the previous (December 1, 2023) CT localization film of the chest (Figure 1: 3B). Therefore the patient was given moxifloxacin (0.4 g qd by intravenous infusion) combined with doxycycline (0.1 g bid PO), and the meropenem was stopped. The patient’s condition gradually stabilized under these treatments; methylprednisolone was reduced; and eventually—after 8 days of mechanical ventilation—the tracheal tube was removed to be replaced by a nasal cannula for the administration of oxygen. The bedside chest radiograph was then reviewed again; it pointed to infections in both lungs, similar to the previous one (December 6, 2023), with a small pleural effusion on the left side (Figure 1: 3C). After extubation, the patient appeared to have blood in her sputum. A review of the color Doppler ultrasound of her lower extremity vessels suggested that there was no increase in thrombus, so the nadroparin calcium was stopped. Three days after extubation, the patient was stable and transferred back to respiratory medicine.

**FIGURE 2:**
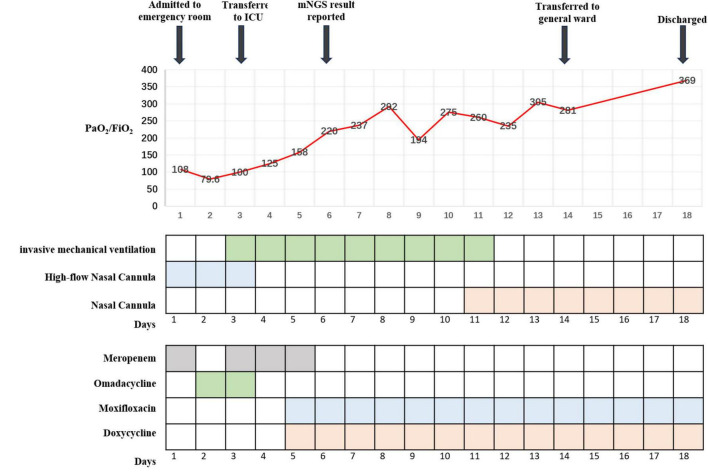
Hospitalization timeline and clinical treatments.

In the respiratory medicine department, moxifloxacin and doxycycline were continued up to 14 days. During this period, CT pulmonary angiography did not indicate a thrombus. A repeat CT scan of the chest suggested partial resorption of the lesions whereas others had increased, as had the bilateral pleural effusions (Figure 1: 4A–C). After 14 days of antichlamydial treatment, the patient’s blood inflammatory index had returned to normal; therefore, the antibiotics were stopped. Because the serum D-dimer level increased to 12.79 μg/mL, after communicating with the patient’s family members, the patient was discharged with a prescription for rivaroxaban 10 mg qd PO. A month after discharge, the patient was reexamined by CT of the chest, indicating significant absorption of both lung lesions and pleural effusions (Figure 1: 5A–C). The full timeline of hospitalization and clinical treatment is shown in Figure 2.

## Discussion

*Chlamydia abortus* used to be classified as a subspecies of *Chlamydia psittaci*, which caused mainly animal infections, with abortion and stillbirth being common among pregnant cows and sheep (8) and could also lead to miscarriage in pregnant women, sepsis in pregnancy, and pelvic inflammatory disease (3, 9, 10). However, thus far, only six cases of human pneumonia due to *C. abortus* have been found worldwide. *C. abortus* organisms can be excreted through urine, feces, lactated milk, amniotic fluid, placenta, aborted fetus, and other routes in diseased animals (11). Based on the findings of previously reported cases, most patients infected with *C. abortus* appear to have a history of direct or indirect contact with infected animals (2–7). This suggests that humans can become ill through direct contact with infected animals or secondary owing to contamination of the environment. The patient in this case was a farmer who fed small animals such as chickens, ducks, and pigs at home and had regular contact with these animals. Therefore we suspected that the patient might have developed pneumonia through direct contact with animals infected with *C. abortus*. No one else in the family was known to have had a similar infection.

*C. abortus* infection initially presents with flulike symptoms, causing fever, headache, and weakness in the limbs. Due to the lack of specificity of the clinical symptoms and clinicians’ low level of knowledge of the disease, it is easy to miss or misdiagnose this disease. Chlamydial infection can also cause serious visceral complications; therefore, clinicians need to be alert to this possibility (12, 13). In this case, the patient presented with typical influenza-like symptoms (such as fever and generalized muscle pain) at the beginning of the disease, followed by respiratory symptoms (such as cough, phlegm, and dyspnea). These were accompanied by extrapulmonary manifestations such as diarrhea as well as laboratory tests suggesting hyponatremia and hypochlorhydria, impaired hepatic and renal function, and elevated lactate dehydrogenase. These are similar to the clinical features of *Legionella* pneumonitis (14) and *C. psittaci* pneumonia (15).

This patient’s lung imaging showed rapid progression from a solid lesion in the upper lobe of the left lung to solid lesions in multiple lobes, with air bronchial signs at the site of the solid lesion accompanied by bilateral pleural effusions. These imaging features are nonspecific and are also seen in streptococcal and other atypical infections, making early diagnosis of the pathogen critical.

Pneumonia caused by *C. abortus* is very rare and cannot be detected by current detection methods, such as specimen culture, serologic detection, and the polymerase chain reaction. mNGS, as an unbiased method—with its high detection speed, high accuracy, wide coverage, and high throughput (16)—theoretically detects all pathogens in clinical samples and is particularly suitable for rare and complex infectious diseases with atypical etiology (17). In this case—after blood culture, sputum culture, and chlamydial nucleic acid testing were all found to be negative—we were able to diagnose *C. abortus* by mNGS.

*C. abortus* is unaffected by beta-lactam antibiotics because it lacks a cell wall. *Chlamydia* is usually sensitive to antibiotics that inhibit DNA and protein synthesis, including tetracyclines, macrolides, and quinolones. Therefore, tetracyclines are used as first-line therapeutic agents, whereas other approaches include macrolides and quinolones (18). The length of antibiotic administration is usually 10–14 days. In this case, immediately after confirmation of *C. abortus* infection, the antibiotics were adjusted to doxycycline combined with moxifloxacin, which had an immediate effect. Repeat CT of the chest 1 month later showed substantial resorption of both lung lesions.

In summary, ruminants can transmit *Chlamydia abortus* to humans. Its early diagnosis is low owing to nonspecific clinical signs and imaging features, making it hard to detect by conventional tests. Therefore, if early β-amide antibiotics are ineffective in patients with pneumonia who have a history of ruminant exposure, the possibility of infection with *Chlamydia abortus* must be considered. mNGS of the bronchoalveolar lavage fluid is an important method for diagnosing this disease.

## Data availability statement

The original contributions presented in this study are included in this article/supplementary material, further inquiries can be directed to the corresponding author.

## Ethics statement

Written informed consent was obtained from the individual(s) for the publication of any potentially identifiable images or data included in this article.

## Author contributions

Q-FY: Writing – original draft, Writing – review and editing. C-MS: Writing – review and editing.
